# Tracing the Diploid Ancestry of the Cultivated Octoploid Strawberry

**DOI:** 10.1093/molbev/msaa238

**Published:** 2020-09-17

**Authors:** Chao Feng, Jing Wang, A J Harris, Kevin M Folta, Mizhen Zhao, Ming Kang

**Affiliations:** 1 Key Laboratory of Plant Resources Conservation and Sustainable Utilization, South China Botanical Garden, Chinese Academy of Sciences, Guangzhou, China; 2 Center of Conservation Biology, Core Botanical Gardens, Chinese Academy of Sciences, Guangzhou, China; 3 Institute of Pomology, Jiangsu Academy of Agricultural Sciences/Jiangsu Key Laboratory for Horticultural Crop Genetic Improvement, Nanjing, China; 4 Department of Biology, Oberlin College, Oberlin, OH; 5 Horticultural Sciences Department, University of Florida, Gainesville, FL

**Keywords:** *Fragaria*, chromosome-level genome, diploid progenitors, gene tree discordance, ILS and hybridization, sppIDer

## Abstract

The commercial strawberry, *Fragaria* × *ananassa*, is a recent allo-octoploid that is cultivated worldwide. However, other than *Fragaria vesca*, which is universally accepted one of its diploid ancestors, its other early diploid progenitors remain unclear. Here, we performed comparative analyses of the genomes of five diploid strawberries, *F. iinumae*, *F. vesca*, *F. nilgerrensis*, *F. nubicola*, and *F. viridis*, of which the latter three are newly sequenced*.* We found that the genomes of these species share highly conserved gene content and gene order. Using an alignment-based approach, we show that *F. iinumae* and *F. vesca* are the diploid progenitors to the octoploid *F.* × *ananassa*, whereas the other three diploids that we analyzed in this study are not parental species. We generated a fully resolved, dated phylogeny of *Fragaria*, and determined that the genus arose ∼6.37 Ma. Our results effectively resolve conflicting hypotheses regarding the putative diploid progenitors of the cultivated strawberry, establish a reliable backbone phylogeny for the genus, and provide genetic resources for molecular breeding.

The commercial strawberry, *Fragaria* × *ananassa*, is one of the most recently domesticated plants in the world and is among many economically important fruit crops of the Rosaceae plant family (Hummer and Hancock 2009). According to the Food and Agriculture Organization (FAO) of the United Nations, world production of strawberries has exceeded 8 million tons since 2018 (FAO 2020). In addition to being visually appealing and tasty, strawberries provide a wide range of nutritional benefits because they are rich in vitamin C, phenolic compounds, and micronutrients (Giampieri et al. 2012).

The genus *Fragaria* is circumscribed with ∼25 species, which represent five ploidy levels, ranging from diploid to decaploid with a base chromosome number of 7 (Folta and Davis 2006; Hummer and Hancock 2009; Lei et al. 2017). Species of *Fragaria* have a natural distribution in the Northern Hemisphere with their center of diversity being within China, where the most diploid (8 out of 12) and all five tetraploid species of the genus occur (Liston et al. 2014; Lei et al. 2017). Wild species of *Fragaria* are known to have small genomes (∼200–300 Mb for diploid species) and diverse breeding systems from self-compatibility to dioecy and, among species, barriers to crossing are low. Most species of *Fragaria* can be clonally propagated by stolons. Mature plants are usually small and this facilitates cultivation in enclosed, controlled conditions. These characteristics render *Fragaria* a uniquely powerful system for studies of sexual system evolution, polyploidization, and evolutionary genomics (Liston et al. 2014). Moreover, wild species are valuable in breeding programs aimed at broadening the gene pools for cultivated strawberries (Hancock 1999; Chambers et al. 2013).

The modern cultivated strawberry is a recent allo-octoploid (2*n* = 8*x* = 56) species, which is thought to have arisen via spontaneous hybridization between representatives of its two octoploid progenitor species, *Fragaria chiloensis* and *F. virginiana*, in Europe in the mid-18th century (Darrow 1966). To elucidate the precursory diploid progenitors, numerous cytological and phylogenetic studies have been undertaken and have led to four contradictory hypotheses involving two to five plausible diploid progenitors for the octoploid genome (Fedorova 1946; Senanayake and Bringhurst 1967; Bringhurst 1990; Rousseau-Gueutin et al. 2009; DiMeglio et al. 2014; Tennessen et al. 2014; Sargent et al. 2016; Kamneva et al. 2017; Yang and Davis 2017). For example, Tennessen et al. (2014) hypothesized that the allo-octoploid cultivated strawberry *F.* × *ananassa* originated from a complex series of genetic contributions from *F. vesca*, *F. iinumae*, and two *F. iinumae-*like ancestors, based on evidence from linkage maps. In contrast, Yang and Davis (2017) proposed that genetic signatures of at least five diploid ancestors (*F. vesca*, *F. iinumae*, *F. bucharica*, *F. viridis*, and one with an unknown identity) are present in octoploid *Fragaria* species. Recently, Edger et al. (2019) proposed that four diploid species, *F. vesca*, *F. iinumae*, *F. viridis*, and *F. nipponica*, comprise the subgenomes of the octoploid strawberry, *F.* × *ananassa*, based on a high-quality, sequenced genome of the commercial species and a tree-searching algorithm. They further suggested that the hexaploid species *F. moschata* may be evolutionary intermediate in the formation of the octoploid species. However, this hypothesis was rejected with a reanalysis of these data (Liston et al. 2020). Thus, although *F. vesca* has been universally accepted as a diploid ancestor in previous studies, the subgenomic composition of the octoploid strawberry is still under debate (Edger et al. 2020; Liston et al. 2020).

Understanding the phylogenetic relationships among species of *Fragaria* is critical for unraveling the diploid origins of this crop. However, the relationships within this genus remain recalcitrant to phylogenetic resolution (Liston et al. 2014), especially the position of three diploid species, *F. viridis*, *F. nilgerrensis*, and *F. iinumae*, even using sizable, multilocus gene data sets (Qiao et al. 2016; Kamneva et al. 2017; Yang and Davis 2017). Inferring the correct species phylogeny for recently diverged lineages such as *Fragaria* is notoriously challenging because both incomplete lineage sorting (ILS) and hybridization often cause discordance between gene and species trees. Therefore, phylogenetic methods can potentially result in misleading conclusions. Moreover, hybrid species such as *F.* × *ananassa* specifically violate some fundamental assumptions of phylogenetic methods, so integrating these species into molecular phylogeny to determine their closeness to putative parental species may be problematic. To overcome this, sppIDer (Langdon et al. 2018) was recently developed for mapping short-read sequencing data to a composite reference genome constructed from potential progenitor species to determine their contributions to hybrid genomes. This method does not require the underlying assumptions of phylogenetic methods and can, therefore, mitigate their drawbacks. SppIDer has been shown to have high accuracy in identifying the genomic origins of hybrid species (Langdon et al. 2018).

Here, we fully sequenced and assembled de novo genomes of three wild, diploid strawberry species: *F. nilgerrensis*, *F. nubicola*, and *F. viridis*. Combined with the existing high-quality assemblies of *F. vesca* (Edger et al. 2017) and *F. iinumae* (Edger et al. 2020), we performed comparative genomic analyses that revealed a high degree of conserved genomic composition across species and only a small number of species-specific genes. Using these five genomes, we reconstructed a phylogeny of *Fragaria* and inferred the crown age of the genus as ∼6.37 Ma. The phylogeny exhibits high levels of gene tree discordance due to both extensive ILS and interspecific hybridization. Finally, using sppIDer, we clearly show that *F. iinumae* and *F. vesca* are the diploid progenitors of the octoploid *F.* × *ananassa*, whereas *F. viridis* is not a parental species. Our analyses provide new insights into the evolutionary history of *Fragaria* and resolve the origins of the commercial species.

## Results and Discussion

### The Conserved Genome across the Five Diploid Species

**Fig. 1:**
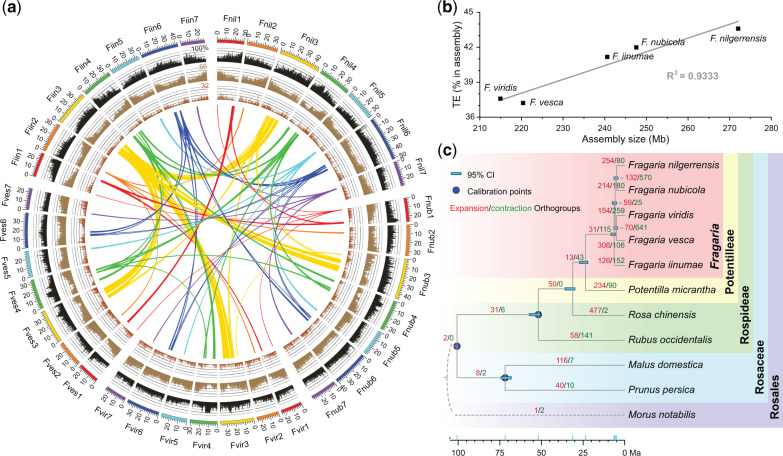
Features of five diploid *Fragaria* genomes and evolution across Rosaceae. (*a*) Multidimensional topography for *F. nilgerrensis* (*Fnil*), *F. nubicola* (*Fnub*), *F. viridis* (*Fvir*), *F. vesca* (*Fves*), and *F. iinumae* (*Fiin*) genomes. Circos plots as concentric circles from outermost to innermost show TE percentage (dark gray columns), gene density (Brown columns), density of tandem duplicates (red-brown columns), and inverted regions detected among the assemblies of the five genomes (colorful links). Details of syntenic blocks are provided in supplementary figures S5 and S6, Supplementary Material online. Each column represents a 250-kb nonoverlapping window, and every link contains at least 100 genes. (*b*) Scatter plot and regression showing significant positive correlations between TE proportion and assembly size. (*c*) Phylogenetic tree showing the topology, divergence time, and expansions/contractions of orthologous groups for ten species of Rosaceae. The species tree was constructed by ASTRAL using 1,476 single copy orthologous groups with *Morus notabilis* as the outgroup. The divergence time was estimated by r8s according to three node age calibrations (blue circles). Blue bars indicate the 95% confidence intervals (CI) of divergence times. Red and green numbers along the branches show expanded and contracted orthologous groups, respectively.

We adopted PacBio (Pacific Biosystems) long-read sequencing (104–116× coverage) and Hi-C (High-throughput Chromosome Conformation Capture) technologies to sequence and assemble chromosome-level genomes for three diploid species of *Fragaria* (2*n* = 2*x* = 14): *F. nilgerrensis*, *F. nubicola*, and *F. viridis* (fig. 1*a*;supplementary fig. S1 and table S1, Supplementary Material online). The length of the assemblies ranged from 214.6 to 272.0 Mb with N50 of contigs between 2.5 and 4.0 Mb, and 97.1–98.2% of contigs being anchored to seven pseudomolecules (table 1; supplementary tables S2 and S3, Supplementary Material online). We determined that the assemblies exhibited high levels of completeness and consistency through a series of assessments (supplementary figs. S2–S4 and tables S4–S9; supplementary methods, Supplementary Material online). Comparisons among assemblies and to the available genomes of *F. vesca* (Edger et al. 2017) and *F. iinumae* (Edger et al. 2020) revealed significant positive correlations between the proportion of transposable elements (TEs) and assembly size, indicating that size variation in the diploid genomes is mainly driven by TE proliferation (fig. 1*b*;supplementary tables S3 and S9, Supplementary Material online).

**Table 1:** Summary Statistics from the Assembly and Annotation of Three Diploid Species of *Fragaria*.

	*Fragaria nilgerrensis*	*Fragaria nubicola*	*Fragaria viridis*
Assembly feature			
Genome-sequencing depth (×)	373	406	473
Estimated genome size (Mb)	279	273	219
Total length of scaffolds (Mb)	272.0	247.5	214.9
N50 of scaffolds (Mb)	37.5	35.0	29.2
Total length of contigs (Mb)	271.9	247.2	214.6
N50 of contigs (Mb)	4.0	2.6	3.5
Mapping rate of reads from short-insert libraries	96.3%	90.0%	94.3%
CEGMA evaluation	97.2%	92.3%	94.8%
BUSCO evaluation	93.7%	87.5%	88.5%
LAI evaluation	10.2	17.5	16.7
EST evaluation	92.5%	92.4%	93.2%
RNA-Seq evaluation	88.6–93.4%	81.0–84.4%	82.7–86.6%
Genome annotation			
Percentage of TE	43.60	43.07	38.67
Percentage of LTRs	35.29	32.87	26.92
No. of predicted protein-coding genes	29,068	27,594	26,199
No. of genes annotated to public database	26,353	25,418	24,370

Using a combination of de novo identification, homology-based prediction, and RNA-Seq-based prediction, we identified 26,199–29,068 protein-coding genes in the newly assembled genomes (supplementary table S10, Supplementary Material online). These gene counts were similar to 28,588 in *F. vesca* (Edger et al. 2017), but slightly higher than 23,665 in *F. iinumae* (Edger et al. 2020). Further, pairwise comparisons among all five genomes revealed that 82.7–91.4% of genes have conserved order, whereas rearrangements occurred for an average of 7.4% of genes (ranging from 1.4% to 13.5%) (fig. 1*a*;supplementary figs. S5 and S6 and table S11, Supplementary Material online). Remarkably, *F. iinumae* has a species-specific inversion (located from 13.4M to 32.7M in chromosome [Chr] 3) that is nearly 20 Mb in length and bears over 1,000 orthologous genes (fig. 1*a*;supplementary fig. S6, Supplementary Material online). Nevertheless, our results suggest highly conserved gene content and gene order across species.

### Young Age of *Fragaria*

The phylogenetic relationships in the genus *Fragaria* have remained controversial and unresolved. We estimated a species tree of *Fragaria* via the summary-coalescent method implemented in ASTRAL (Mirarab and Warnow 2015) using 1,476 single-copy orthologs, which we identified from the Rosaceae family. In this species tree, the phylogenetic relationships among the five diploid species of *Fragaria* were fully resolved with high support (fig. 1*c*). The recovered topology of our species tree is similar to that inferred from 257 genes sequenced by target-capture in a prior study (Kamneva et al. 2017). The only difference is the position of *F. iinumae*. Although *F. iinumae* was nested in a clade with *F. nubicola* in the prior study (Kamneva et al. 2017), we found it to be the first-diverging lineage among the five species.

The topology of our species tree was totally inconsistent with the phylogenetic relationships inferred from a concatenated data matrix of 276 single-copy genes from transcriptome sequencing in Qiao et al. (2016). In that study, the concatenated analysis likely resulted in incorrect phylogenetic relationships among species because phylogenetic reconstruction based on concatenated sequence data cannot account for gene tree heterogeneity (Maddison and Wiens 1997; Degnan and Rosenberg 2009). Given the prevalence of gene tree discordance in *Fragaria* (see below), our inference of a species tree using summary-coalescent methods likely represents a more robust phylogeny of the genus.

Using a well-resolved species tree representing Rosaceae, we dated the origin of the crown node of *Fragaria* to be 6.37 Ma (95% CI: 5.54–8.38 Ma; fig. 1*c*). This age is substantially older than that previously inferred with chloroplast genomic data (1.52–4.44 Ma; Njuguna et al. 2013). This difference may be because chloroplast genes evolve slowly (Wolfe et al. 1987). In addition, we used a more ancient fossil, *Prunus wutuensis* (age: Early Eocene, 55.0 Ma), to calibrate the stem node of *Prunus* (Xiang et al. 2017) compared with Njuguna et al. (2013), who used *Prunus cathybrownae* (age: late Early Eocene, 48.4 Ma; Benedict et al. 2011). Although the 95% credibility interval from this study is largely overlapping with that estimated from transcriptomic data (Qiao et al. 2016), our estimate of the median age is slightly younger (i.e., compared with ∼7.99 Ma). Nevertheless, these two dating analyses both suggest that *Fragaria* is a recently diverged lineage, and this may partly explain the conserved genomic structure across species.

### Widespread ILS and Hybridization across Diploid Genomes

**Fig. 2:**
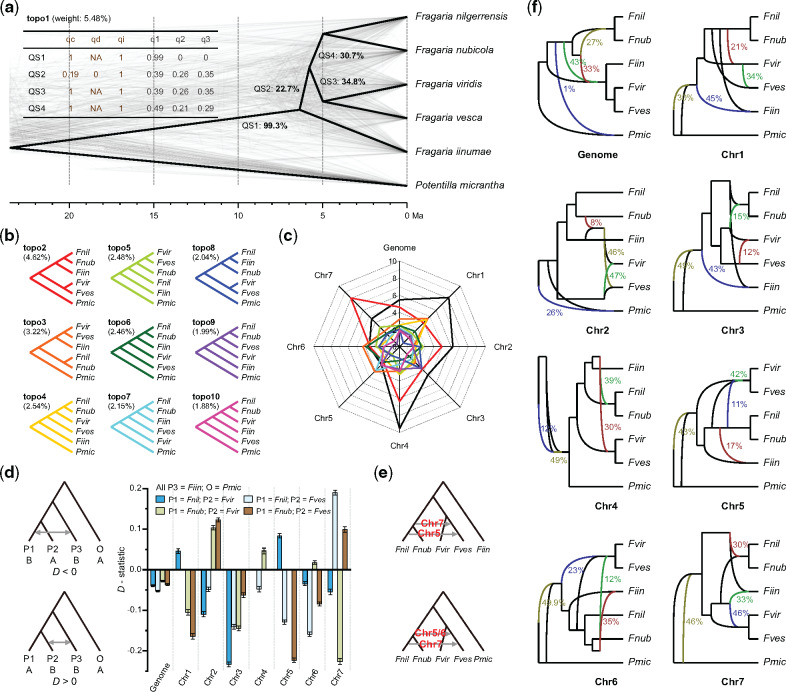
Discordance between gene and species trees, ILS, and gene flow in *Fragaria*. (*a*) Cloudogram of gene trees constructed from 8,663 single copy genes. The topology of the species tree is displayed in bold black, whereas gene trees are plotted in gray. The bold black numbers along the branches indicate the percentage of gene trees that contain each of the subtrees of the species tree. The numbers in brackets indicate the topological weight, or the percentage of gene trees that are consistent with the species tree (i.e., topo1). The brown and gray numbers in the table represent the QS scores and quartet scores from ASTRAL, respectively. (*b*) Nine alternative topologies (topo2–10) for the six sampled taxa, sorted by frequency of occurrence, as shown in brackets. (*c*) Weights for the top ten possible topologies among the whole genome and each chromosome. The black line represents topo1, whereas the colored lines represent the corresponding topologies in (*b*). (*d*) Signals of gene flow in *Fragaria* for the whole genome and each chromosome inferred by the analysis using the *D-*statistic. Columns with different colors indicate the *D*-value for the corresponding four-taxon phylogeny with *Z* value higher than 3.0. Negative and positive *D*-values represent the strength of gene flow between P1 and P3 and between P2 and P3. The results of the *D-*statistic analysis for all combinations of a four-taxon phylogeny are shown in supplementary table S13, Supplementary Material online. (*e*) Signals of gene flow in *Fragaria* inferred by *D*_FOIL_ analyses. Details are provided in supplementary table S14, Supplementary Material online. (*f*) Signals of gene flow in *Fragaria* among the whole genome and each chromosome inferred by Bayesian MCMC posterior estimation in PhyloNet. Olive, green, dark red, and blue lines indicate the optimal networks of hybrid nodes H1–4, respectively. Colored numbers next to the lines indicate inheritance probabilities for corresponding edges.

To assess inherent conflicts between gene and species trees for *Fragaria*, we estimated both individual gene trees and a species tree based on 8,663 orthologs shared among the five available diploid genomes and the outgroup, *Potentilla micrantha* (Buti et al. 2018) (fig. 2*a*). Only 5.48% of these gene trees (topo1) were consistent with the species tree, and these also coincided with the phylogenetic position of *F. iinumae* obtained using Quartet Sampling (QS) scores (fig. 2*a*), albeit with weak support. The second and third most frequent topologies (topo2 and topo3, accounting for 4.62% and 2.54% of trees, respectively) show *F. iinumae* as sister to a clade of *F. nilgerrensis* and *F. nubicola*, and a clade of *F. viridis* and *F. vesca*, respectively (fig. 2*b*;supplementary table S12, Supplementary Material online). Remarkably, we found genes of Chr 1–4 more frequently yielded topo1, ranging 4.65–9.53%, whereas genes of Chr 7 more often yielded topo2 (8.04%), and genes of Chr 5–6 more frequently resulted in topo3 (fig. 2*a*–*c*; supplementary table S12, Supplementary Material online). These results not only demonstrate widespread gene tree discordance across the *Fragaria* genome but also suggest unique evolutionary histories for each chromosome.

To further dissect the cause of the phylogenetic discordance, we assessed the degree of ILS across the genus according to ASTRAL quartet scores (Mirarab et al. 2014). All branches except that subtending the crown node of *Fragaria* (QS2–4) have low major quartet scores (q1) of <0.5 (fig. 2*a*), indicating high levels of ILS (Mirarab et al. 2014). Branches QS2 and QS3 received almost equal quartet scores for q1, q2, and q3 (fig. 2*a*), suggesting that the gene trees yield random topologies with respect to the species tree, and levels of ILS are extremely high.

We also identified signals of hybridization by using a combination of the *D*-statistic (Durand et al. 2011), *D*_FOIL_ (Pease and Hahn 2015), and PhyloNet (Wen et al. 2018) analyses. The *D*-statistic showed significant gene flow between *F. iinumae* and both *F. nilgerrensis* and *F. nubicola* at the whole-genome level (fig. 2*d*;supplementary table S13, Supplementary Material online), and this could explain the phylogenetic relationships of topo2, in which *F. iinumae* is sister to the clade of *F. nilgerrensis* and *F. nubicola* (fig. 2*b*). Notably, we also detected numerous signals of gene flow between *F. iinumae* and both *F. viridis* and *F. vesca* based on four-taxon phylogenies constructed using genes of individual chromosomes (fig. 2*d*;supplementary table S13, Supplementary Material online). Overall, the results indicate a complex pattern of gene flow among species of *Fragaria*.

We performed *D*_FOIL_ analyses to evaluate two alternative topologies for each of the seven chromosomes of *Fragaria*. For Chr 5, 6, and 7, we found a strong signal for gene flow from *F. nilgerrensis* or the most recent common ancestors of *F. nilgerrensis* and *F. nubicola* to *F. vesca* (fig. 2*e*;supplementary table S14, Supplementary Material online), and this is in agreement with the inconsistent topologies of Chr 5, 6, and 7 compared with other chromosomes. Similarly, PhyloNet identified extremely complicated and statistically significant signals for gene flow across the genus and showed that signals of introgression vary greatly among different chromosomes (fig. 2*f*). Collectively, our results suggest that *Fragaria* is especially prone to hybridization. Our findings of the prevalence of both ILS and hybridization in the genus agree with those from other recently diverged lineages (Novikova et al. 2016; Liu et al. 2018; Wu et al. 2018) and highlight the roles of both of these mechanisms in shaping genomic and species evolution.

### Tracing the Diploid Ancestors of the Cultivated Strawberry

Widespread ILS and hybridization across genomes in *Fragaria* make it difficult to trace the diploid origins of the octoploid strawberry. Previous work based on phylogenetic approaches has led to conflicting hypotheses (Rousseau-Gueutin et al. 2009; Tennessen et al. 2014; Sargent et al. 2016; Kamneva et al. 2017; Yang and Davis 2017; Edger et al. 2019, 2020; Liston et al. 2020). Although *F. vesca* and *F. iinumae* have been commonly proposed as ancestors, which/whether additional subgenomes contributed to the octoploid genome remains under debate (Edger et al. 2019, 2020; Liston et al. 2020). Specifically, *F. viridis* and *F. nipponica* were identified as two additional, putative ancestors based on a tree-searching algorithm (Edger et al. 2019). However, reanalysis of the same data sets using a chromosome-scale phylogenomic approach led Liston et al. (2020) to argue that unsampled or an extinct populations of *F. iinumae* comprise the progenitors of *F.* × *ananassa*.

**Fig. 3:**
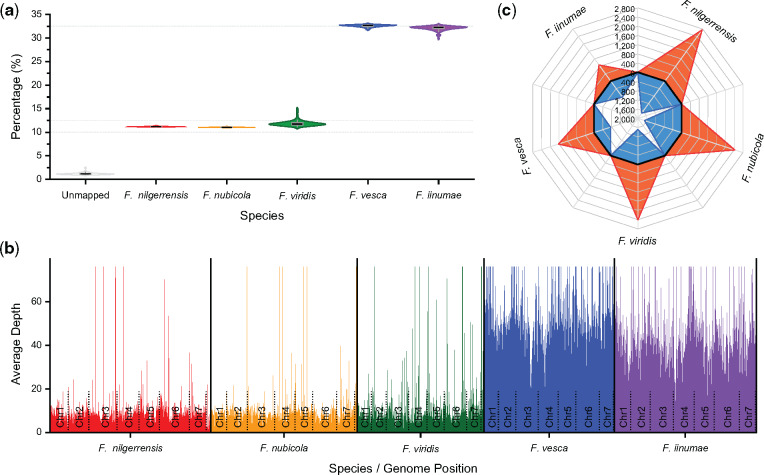
Tracing the diploid ancestors of the cultivated octoploid strawberry.(*a*) Comparison of the percentage of reads from 73 octoploid strawberries, *F.* × *ananassa* genotype resequencing data (downloaded from NCBI PRJNA578384), mapped to the five diploid species of *Fragaria*. (*b*) Visual example of depth of mapped reads of a representative octoploid strawberry, FL_13C026p134 (accession numbers: SRR10312160 and SRR10312161), across the composite genomes of putative diploid progenitors. Details are provided in supplementary figure S7, Supplementary Material online. (*c*) Comparison of the number of genes that exist in diploid species of *Fragaria* but lack orthologs in *F.* × *ananassa*. The orange regions indicate the number of species-specific genes in five diploid species of *Fragaria*, whereas the blue regions represent the number of genes belonging to the orthologous groups shared in two or more diploid species of *Fragaria* but absent in cultivated strawberry.

To avoid the drawbacks arising from phylogenetic approaches, we applied a novel alignment-based approach, sppIDer (Langdon et al. 2018), which directly maps short-read sequence data to a composite reference genome constructed from potential progenitors to determine their contributions to hybrid genomes. Using this method, we mapped sequence data from 73 genomes of the octoploid strawberry, *F.* × *ananassa* (supplementary table S15, Supplementary Material online), to a composite of five diploid genomes of *Fragaria*. We found similar trends for each of the 73 octoploid genomes; namely that nearly one-third of reads were mapped to *F. vesca* and *F. iinumae*, whereas only ∼12%, 11%, and 11% of reads were mapped to *F. viridis*, *F. nilgerrensis*, and *F. nubicola*, respectively (fig. 3*a*). Furthermore, we found that mapped reads of a representative genome of *F.* × *ananassa* across the composite genome were relatively consistent in coverage depth, with only a few loci containing aberrant coverage that could be consistent with introgression (fig. 3*b*;supplementary fig. S7, Supplementary Material online). These results suggest that *F. vesca* and *F. iinumae*, rather than *F. viridis*, *F. nilgerrensis*, and *F. nubicola*, are the diploid progenitors of the octoploid commercial strawberry. This finding is in agreement with the results of Liston et al. (2020) but rejects the hypothesis of Edger et al. (2019, 2020), who found that *F. viridis* represented one of the four subgenomes of the cultivated strawberry. However, at present, we cannot entirely rule out genomic contributions from other diploid species to the cultivated strawberry, and high-quality genomes from additional diploid *Fragaria* are needed to fully confirm our hypothesis.

We further compared proteins from the cultivated octoploid strawberry with those in the five diploid species of *Fragaria*. We found that *F. iinumae* and *F. vesca* have only 1,726 and 2,383 genes, respectively, that have no orthologs in *F.* × *ananassa*, whereas the other three diploid species have a much larger number of genes absent from the commercial strawberry (3,929–4,466; fig. 3*c*;supplementary fig. S8, Supplementary Material online). These proteomic data provide additional evidence that *F. iinumae* and *F. vesca*, not *F. viridis, F. nubicola* and/or *F. nilgerrensis*, are the diploid progenitors of *F.* × *ananassa*.

Notably, genes that exist in diploid species of *Fragaria* but are absent from cultivated strawberries largely comprise transcription factors (TFs), resistance (R) genes, protein kinases (PKs), and genes related to flowering time (such as FT) and fruit quality, including color, taste, texture, and aroma (supplementary tables S16 and S17, Supplementary Material online). Therefore, the diploid genomes of *Fragaria* provide an extremely valuable resource for identifying genes and alleles for potential genetic improvements to commercial strawberries.

## Materials and Methods

### Genome Sequencing, Assembly, and Annotation

For genome sequencing, we collected the fresh leaves and stolons from one mature individual each of *F. nilgerrensis*, *F. nubicola*, and *F. viridis* in Hubei (111°94′E, 31°76′N), Xizang (88°91′E, 27°71′N), and Xinjiang (83°42′E, 43°22′N) provinces of China, respectively. These germplasm accessions were maintained in the China National Germplasm Repository for Peach and Strawberry (Nanjing) at the Jiangsu Academy of Agricultural Sciences (Nanjing, China).

We assembled the three *Fragaria* genomes according to PacBio SMRT sequencing and Hi-C technology, followed by screened the repetitive sequences and predicted the protein-coding gene structure. Further, we annotated the gene functions of the three *Fragaria* assemblies according to a serious of public databases and identified tandemly repeated gene arrays using TD_identification (Feng et al. 2020).

### Syntenic Analysis among Five *Fragaria* Species

We identified syntenic blocks and generated dot plots for all pairs of the five diploid *Fragaria* species in MCScan (https://github.com/tanghaibao/jcvi/wiki/). Further, we displayed the links of the blocks with CIRCOS (Darzentas 2010).

### Orthogroup Clustering, Species Tree Construction, and Divergence Time Estimation

We used OrthoFinder (Emms and Kelly 2015) to classify the proteins from the five diploid species of *Fragaria* and six other sequenced Rosales plants, including *Potentilla micrantha* (Buti et al. 2018), *Rosa chinensis* (Raymond et al. 2018), *Rubus occidentalis* (VanBuren et al. 2018), *Malus domestica* (Daccord et al. 2017), *Prunus persica* (International Peach Genome Initiative 2013), and *Morus notabilis* (He et al. 2013).

We selected proteins of single-copy orthogroups present in *M. notabilis* and in at least 70% of the other ten plants, followed by aligned these proteins (MAFFT; Katoh and Standley 2013), performed CDS conversion (PAL2NAL; Suyama et al. 2006), applied them to reconstructing gene trees (IQ-TREE; Nguyen et al. 2015), and then obtained a species tree representing Rosaceae using ASTRAL (Mirarab and Warnow 2015). We inferred divergence times in r8s (Sanderson 2003), with two fossil and one secondary age calibrations.

### Discordance Assessment and Gene Flow Analyses

We followed Yang and Smith (2014) to infer orthologs shared among the five diploid *Fragaria* species and *P. micrantha*. Then, we produced a cloudogram with DensiTree (Bouckaert 2010) and evaluated discordance between gene trees and species tree across *Fragaria* using ASTRAL (Mirarab and Warnow 2015) and the QS method (Pease et al. 2018). We also classified the gene trees into different topologies and determined the frequency of each topology at the whole-genome and chromosomal levels. In addition, we detected the signals for introgression across *Fragaria* for the whole genome and each chromosome using a combination of *D*-statistic (Durand et al. 2011), *D*_FOIL_ (Pease and Hahn 2015), and PhyloNet (Wen et al. 2018) analyses.

### Tracing the Diploid Ancestors of the Cultivated Strawberry

We downloaded Illumina resequencing data of 73 cultivated octoploid strawberries (*F. × ananassa*) from the NCBI (BioProject accession number PRJNA578384). We mapped short reads from each sample to the composite reference of the five diploid *Fragaria* genomes in sppIDer (Langdon et al. 2018) and calculated the percentage of read mappings to each of the five genomes.

We applied OrthoFinder (Emms and Kelly 2015) to classify the proteins from the five diploid species of *Fragaria* and the cultivated octoploid species. Then, we compared and annotated the genes that exist in diploid *Fragaria* but lack homologs in the cultivated strawberry.

Detailed methods are included in the supplementary methods, Supplementary Material online.

## Supplementary Material

Supplementary data are available at *Molecular Biology and Evolution* online.

## Supplementary Material

msaa238_Supplementary_Data
