# Supplementary material for: Tracing the Diploid Ancestry of the Cultivated Octoploid Strawberry
Source: Mol Biol Evol. 2020 Sep 17;38(2):478–85. doi: 10.1093/molbev/msaa238 (PMC7826170; doi:10.1093/molbev/msaa238)
Supplement: msaa238_Supplementary_Data [file msaa238_supplementary_data.zip › msaa238-suppl_data/Supplementary Methods.docx]

**Supplementary Methods**

**Illumina sequencing and genome size estimation**

We generated 40.8-44.8 G Illumina shotgun sequencing data (PE150) from libraries with insert sizes of around 350 bp, and after quality control using our previously described pipeline, QC_pe (https://github.com/scbgfengchao/QC_pe; Feng et al. 2017), we obtained 32.0-35.1 G clean data for each of three species of *Fragaria*. We used these data to estimate the genome sizes and heterozygosity of these species in gce (ftp://ftp.genomics.org.cn/pub/gce) according to the following steps. First, we constructed the distribution of *K*-mers (17-mer in this study) with read length (*L*) set to 150 (Fig. S1). Thereafter, we directly obtained the genome size and *a_1/2_* (i.e., percentage of heterozygous *K*-mers) under the parameters *m* = 1, *D* = 8, and *H* = 1. Further, we calculated the heterozygosity index (*H*) according to the formula, *H* = *a_1/2_* / *K* / (2-*a_1/2_*), where *K*=17. In this study, we estimated the genome sizes of *F. nilgerrensis*, *F. nubicola*, and *F. viridis* to be 279, 273, and 219 Mb, respectively, with the heterozygosity of 0.28%, 0.69% and 0.54%, respectively.

**PacBio SMRT sequencing, Hi-C library and genome assembly**

We assembled genomes of the three species of *Fragaria* according to a combined approach utilizing PacBio SMRT sequencing and Illumina short reads, to which we applied data from a Hi-C library to anchor the assembly into seven pseudo-molecules. Hi-C and related protocols use proximity ligation and massively parallel sequencing to probe the three-dimensional architecture of chromosomes within the nucleus, with interacting regions captured to paired-end reads. In the resulting data sets, the probability of intrachromosomal contacts is on average much higher than that of interchromosomal contacts (Burton et al. 2013). The details of our assembly protocol are as follows:

**1) Pre-assembly of PacBio reads.** We generated 25.4-29.8 G PacBio SMRT reads (with coverage over 100× for each species) with N50 of Subread length ranging 17.4-18.8 kb (Table S1) for the three species of *Fragaria*. Thereafter, we applied ‘daligner’ from FALCON (https://github.com/PacificBiosciences/FALCON/) to correct errors in the PacBio long reads by the PacBio short reads (<5 kb), and to generate consensus sequences under the parameters *output_multi*, *min_idt* = 0.70, *min_cov* = 4, and *max_n_read* = 200. Further, we detected and filtered overlaps between all pairs of the error-corrected reads in FALCON with parameters *max_diff* = 100, *max_cov* = 100, and *min_cov* = 2, and applied the read overlaps to construct string graphs using Myers’ algorithm (Myers 2005). According to the paths from these graphs, we assembled the primary contigs. Finally, we curated the contigs by aligning SMRT reads in Quiver and by aligning Illumina short reads with Pilon (https://github.com/broadinstitute/pilon).

**2) Removal of incorrectly assembled heterozygous alleles**. We used Purge Haplotigs (Roach et al. 2018) to identify heterozygous alleles incorrectly assembled separately from their allelic counterpart, specifically by seeking contigs with a high proportion of bases with read-depth range of 0.5×. We subjected the candidate heterozygous contigs to sequence alignment to identify haplotigs (i.e., allelic contigs) and artefacts (i.e., the contigs with abnormal coverage) using Mini-map2 (Li 2018) and Purge Haplotigs (Roach et al. 2018). After removing the haplotigs iteratively, we obtained a deduplicated representation of the genome. We repeated this pipeline for several read-depth cutoffs and finally achieved a version that balanced removal of redundancy with integrity. The total deduplicated representations for *F. nilgerrensis*, *F. nubicola*, and *F. viridis* were 271.9 Mb, 247.2 Mb, and 214.6 Mb, respectively, with N50 up to 4.0 Mb, 2.6 Mb and 3.5 Mb, respectively. These final assemblies accounted for 97.5%, 90.7% and 98.1% of the estimated genome sizes of these species, respectively (Table 1, S2).

**3) Anchoring to pseudo-molecules.** We generated 39.1-50.4 G of clean data from Hi-C libraries for the three species of *Fragaria*. After removing low quality Hi-C reads, such as the reads with larger than 10% unidentified nucleotides (N), the ones aligned to the adapter, and the ones with over half of bases having phred quality < 5, we aligned the filtered Hi-C read pairs to the deduplicated PacBio assembly using BWA (Li and Durbin 2009). Further, we removed the reads that were mapped to regions over 500 bp from a restriction site, and only retained uniquely mapped and valid paired-end di-tag reads for subsequent analysis. In addition, to correct errors within the PacBio assembly, we clustered, sorted, oriented and anchored the corrected contigs into seven pseudo-molecules by using ACHESIS (Burton *et al*., 2013) with parameters of *CLUSTER_MIN_RE_SITES* = 22, *CLUSTER_MAX_LINK_DENSITY* = 2, *CLUSTER_NONINFORMATIVE_RATIO* = 2, *ORDER_MIN_N_RES_IN_TRUN* = 10, and *ORDER_MIN_N_RES_IN_SHREDS* = 10. Finally, we achieved three chromosome-level genomes, with 97.1-98.2% of the assembly being anchored to seven pseudo-molecules (Table S3).

**Genome assembly assessment.**

We carried out rigorous assessments of the consistency and completeness of the genome assemblies of the three species of *Fragaria*.

First, we divided the assembly into 100 kb sections and determined the number of Hi-C reads representing links between each pair of adjacent sections. The heat maps of these linkages indicate the expected break-points supporting good-quality assembly of the pseudo-molecules for each of the three species (Fig. S2-S4).

Second, we mapped reads from the Illumina short-insert libraries (~350 bp) to the assembly using BWA (Li and Durbin 2009) and called SNPs using SAMtools (Li et al. 2009). We found that over 99% of regions of the genomes were covered by reads with depth ≥ 10, and the homologous SNP ratio range between 0.0015% and 0.0033% (Table S4). These statistics suggest excellent consistency and accuracy in the assemblies.

Third, we applied two pipelines to evaluate gene space coverage based on RNA-Seq data for several different tissues represented by six to 13 tissue samples for each species (Table S5). Specifically, we directly aligned the RNA-Seq reads to the assembly by Tophat2 (Kim et al. 2013) and we also *de novo* assembled all the RNA-Seq data from the same species into ESTs using Trinity (Grabherr et al. 2011). After removing the ESTs that were not mapped by Illumina reads from DNA, then we aligned the filtered ESTs to the assembly using BLAT (Kent 2002). The results suggested that the genomic assemblies of the three species of *Fragaria* have relatively high completeness (Tables S5, S6).

Fourth, we used several public databases to evaluate the completeness of the gene space. Among these databases were embryophta_odb10, containing 1,375 benchmarking universal single-copy orthologs (BUSCOs) (Waterhouse et al. 2018) and ultra-conserved core Eukaryotic genes (CEG), and we aligned the proteins (identified by Gene structure prediction, see below) of the three genomes of *Fragaria* to both of these. We also calculated the LTR Assembly Index (LAI) for each assembly using LTR_retriever (Ou and Jiang 2018) and the resulting values suggested that all three assemblies of *Fragaria* were relatively robust (Table S7-S9).

**Repetitive sequences annotation**

We screened the repetitive sequences in five genomes of *Fragaria* using a combination of *de novo* identification and homology-based prediction at the DNA and protein levels. At the DNA level, we applied RepeatMasker (http://www.repeatmasker.org/) to identify interspersed repeat elements according to a combined library of personalized repeat database and the public Repbase database (<http://www.girinst.org/repbase/>). The personalized repeat database for each of five species of *Fragaria* was constructed *de novo* using LTR_FINDER (Xu and Wang 2007), Piler (http://www.drive5.com/piler/) and RepeatModeler (http://www.repeatmasker.org). At the protein level, we used RepeatProteinMask (http://www.repeatmasker.org) to detect interspersed repeat elements against the Repbase database. Overall, transposable elements (TEs) accounted for 43.60%, 41.99%, 37.59%, 37.22%, and 41.18% of the genomes of *F. nilgerrensis*, *F. nubicola*, *F. viridis*, *F. vesca*, and *F. iinumae*, respectively (Table S9).

**Gene structure prediction.**

We predicted the protein-coding gene sequence (i.e., linear) structure using a combination of *de novo* identification, homology-based prediction, and RNA-Seq-based prediction.

For *de novo* identification, we used Augustus (Stanke et al. 2006), Geneid (Guigó et al. 1992), SNAP (Korf 2004), GlimmerHMM (Majoros et al. 2004), and Genscan (Burge and Karlin 1997).

For homology-based prediction, we obtained the proteins from several sequenced model plants, i.e., *Fragaria vesca*, *Malus domestica*, *Prunus persica*, *Solanum lycopersicum*, *Arabidopsis thaliana*, and *Vitis vinifera*, and aligned these to each of the three newly assembled genomes of the species of *Fragaria* using tBlastN (Altschul et al. 1990). According to the alignment, we generated the gene models in Genewise (Birney et al. 2004).

For RNA-Seq-based prediction, we applied two pipelines. One pipeline consisted of using cufflinks (Trapnell et al. 2010) to determine the gene structures according to the alignment of RNA data against the assembly. The other pipeline comprised applying PASA (Haas et al. 2003) to generate the gene structures from the alignment of ESTs of each species to the assembly.

We generated a composite model of gene structure from the above three approaches using a combination of EVM (Haas et al. 2008) and PASA. After removing genes that were only predicted via *de novo* identification, we constructed non-redundant models of gene structure for each species. In total, there were 29,068, 27,594, and 26,199 protein-coding gene models for *F. nilgerrensis*, *F. nubicola*, and *F. viridis*, respectively (Table S10).

**Identification of tandem repeats**

We identified tandem duplications (i.e., tandemly repeated gene arrays) using TD_identification (https://github.com/scbgfengchao/TD_identification; Feng et al. 2020) with default parameters. Based on this method, we detected 1,191-1,509 tandem groups comprising 3043-3,962 genes in the five diploid species of *Fragaria* and visualized their distribution among the chromosomes via CIRCOS (Darzentas 2010) (Fig. 1a).

**Syntenic analysis**

We constructed syntenic blocks and generated dot plot graphs for these for all pairs of the five species of *Fragaria* (*F. nilgerrensis*, *F. nubicola*, *F. viridis*, *F. vesca* and *F. innumae*) using MCScan (<https://github.com/tanghaibao/jcvi/wiki/>) (Fig. S6; Table S11). Further, we displayed organization of the blocks containing over 100 genes among the genomes via CIRCOS (Darzentas 2010) (Fig. 1a, Fig. S5).

**Gene function annotation**

We annotated the functions of genes of the three newly sequenced species of *Fragaria* by utilizing several public databases. We first assessed predicted proteins using the TrEMBL, Swissprot, and KEGG databases. In addition, we applied InterProScan (Zdobnov and Apweiler 2001) to predict the domains of genes according to the InterPro Consortium (http://www.ebi.ac.uk/interpro/), including Pfam (Mistry and Finn 2007), PRINTS (Attwood *et al*., 1994), ProDom (Bru et al. 2005), PROSITE (Hulo et al. 2006), and SMART (Schultz et al. 1998). On a basis of the InterPro result, we generated annotations using Gene Ontology (GO). Overall, we successfully annotated 90.4-92.9% of the proteins comprising the genomes of the three species of *Fragaria* (Table S16).

**Orthogroup clustering and species tree construction**

We classified the proteins from the five diploid species of *Fragaria* and the six other sequenced Rosales plants, including *Potentilla micrantha*, *Rosa chinensis*, *Rubus occidentalis*, *Malus domestica*, *Prunus persica*, and *Morus notabilis*, by using OrthoFinder (Emms and Kelly 2015) under the parameters *S* = diamond and *og*. Further, we compared the core orthogroups among these species and identified that *Fragaria*, Potentilleae (*Fragaria* + *P. micrantha*), Rospideae (Potentilleae + *R. chinensis + R. occidentalis*) and Rosaceae (Rospideae + *M. domestica + P. persica*) , shared 12,740, 11,779, 10,253 and 9,753 orthogroups, respectively (Fig. 1c).

Additionally, we selected proteins of single-copy orthogroups present in *M. notabilis* and in at least 70% of the ten other species. We aligned each of these single-copy orthogroups via MAFFT (Katoh and Standley 2013) and converted them into aligned CDS using PAL2NAL (Suyama et al. 2006). We used the aligned CDS to construct gene trees in IQ-TREE (Nguyen et al. 2015), allowing the program to determine the best substitution model as part of the analysis. Following the analysis, we constructed the species tree in ASTRAL (Mirarab and Warnow 2015), setting *M. notabilis* as the outgroup and using all gene trees of orthogroups excluding partition-specific substitution rates smaller than 0.5 or larger than 2.0.

**Divergence time estimation**

We estimated the species divergence time in r8s (Sanderson 2003) with the *smooth* parameter set to 0.001 and using two fossil and one secondary node age calibrations. For assigning the fossil calibrations, we followed Xiang et al. (2016) in placing the fossil *Prunus wutuensis* (age: late Early Eocene, minimum age of 55.0 Ma) at the stem *Prunus* and placing the fossil *Rubus acutiformis* (age: Middle Eocene, minimum age of 41.3 Ma) at the stem *Rubus*. For the secondary calibration, we dated the divergence of Rosoideae and Amygdaloideae at 100.7 Ma according to Xiang et al. (2016). Further, we applied the r8s_CI pipeline (https://github.com/scbgfengchao/r8s_CI; Feng et al. 2020) to calculate the 95% confidence intervals (CIs) for each node based on 5,000 repeated samplings of proteins in which we randomly sampled 5% of the single-copy orthogroups.

**Orthogroup expansions and contractions**

Using the number of genes per orthogroup in each species according to Orthofinder (Emms and Kelly 2015) (see steps above), we investigated expansion and contractions of gene families along each branch of the dated ultrametric species tree resulting from r8s (Randerson 2003) in CAFE (De Bie et al. 2006). For each gene family, we computed the *p*-values (< 0.01) representing the likelihood of the observed, modern family sizes given the inferred rates of gain and loss.

**Inference of ortholog**

We followed the pipeline of Yang and Smith (2014) (<https://bitbucket.org/yangya/phylogenomic_dataset_construction>) to infer orthologs. Initially, we used MAFFT (Katoh and Standley 2013) to align each orthogroup that was present in all of the ingroup species, including the five diploid species of *Fragaria*, *P. micrantha*, *R. chinensis*, and at least one of the outgroup species (i.e., *R. occidentalis*, *M. domestica*, *P. persica*, and *M. notabilis*). Then we performed two rounds of maximum-likelihood phylogenetic analysis in RAxML (Stamatakis 2014), removing the phylogenies with branches greater than 0.5, and trimmed the ones tips longer than 0.2 or more than 10 times longer than its sister. Based on the remaining gene phylogenies, we determined the 1:1 orthologs (i.e., the orthologs that contain only one gene for each species) using the RT method (Yang and Smith 2014) with complete representation of all ingroup taxa. This pipeline yielded 9,703 1:1 orthologs.

We aligned proteins sequences of the 1:1 orthologs in MAFFT (Katoh and Standley 2013) and converted them into aligned CDS using PAL2NAL (Suyama et al. 2006). We used the aligned CDS to construct gene trees in IQ-TREE (Nguyen et al. 2015) with *R. chinensis* as the outgroup. We retained orthologs with partition-specific substitution rates ranging from 0.5 to 2.0, ultimately yielding a final set of 8,663 1:1 orthologs for subsequent analyses.

**Assessment of discordance between gene trees and the species tree**

For the gene trees representing the 8,663 orthologs, we pruned *R. chinensis* and converted the trees to ultrametric in r8s (Sanderson 2003) using *smooth* = 0.01 and a secondary node age calibration of 23.52 on the stem of *Potentilla*. Using all of the resulting ultrametric trees, we produced a cloudogram of topologies representing the tribe Potentilleae in DensiTree (Bouckaert 2010).

We calculated the percentage of the 8,663 gene trees that contained each of subtrees within the species tree in ASTRAL (Mirarab and Warnow 2015). We also used obtained ASTRAL quartet scores (q1, q2, q3) for each node of the species tree based on the gene trees. A high quartet score of > 0.6 for the resolution observed in the species tree (q1) indicates a low level of incomplete lineage sorting (ILS), while a low score of < 0.5 indicates high level of ILS. Nearly equal portions of resolutions (q1, q2, q3 = ~0.33) indicates extremely significant ILS (Mirarab et al. 2014).

We also applied the Quartet Sampling (QS) method of Pease et al. (2018) to evaluate discordance. For each node of the species tree, this software generates three scores: Quartet Concordance (*qc*), Quartet Differential (*qd*), and Quartet Informativeness (*qi*) (Fig. 2a). Respectively, these scores indicate the support for the node among the gene trees based on analysis of quartets, whether certain alternative quartets (i.e., not represented in the gene tree) around each node occurs more often than others, and the informativeness of quartets around each node. A QS result of *qc* = 1, *qd* = NA, and *qi* = 1 indicates that all gene trees support the node on the species tree, while scores such as *qc* = 0.19, *qd* = 0, and *qi* = 1 would indicate weak support for the node on the species tree and show that there are alternatives for that node disproportionately represented in the gene tree data.

We used topological weighting to reduce the complexity of the six-taxon phylogenies of tribe Potentilleae (i.e., five species of Fragaria plus *P. micrantha* as the outgroup). Ignoring the branch lengths, there are three possible tree shapes for the six-taxon phylogeny: (i), (((((A,B),C),D),E),F); (ii), ((((A,B),(C,D)),E),F); and (iii), ((((A,B),C),(D,E)),F) where taxon F is always the outgroup but A-E may be any member of the ingroup. Thus, the total number of possible topologies can be calculated as: N_i_ = $∁_{5}^{2}\times A_{3}^{3}$= 60; N_ii_ = $∁_{5}^{2}\times∁_{3}^{2}\times∁_{1}^{1}$= 30; N_iii_ = $∁_{5}^{2}\times∁_{3}^{1}\times∁_{2}^{2}$= 30. Therefore, there are a maximum to 120 possible topologies for the six taxa. We determined that the 8,663 gene trees could be classified into 105 topologies, for which we calculated the frequency as a weight. We also determined weights of the alternative topologies for the genes on each of the seven chromosomes (Fig. 2a, b, c; Table S12).

**Analyses of gene flow**

We detected the signals for introgression among species of *Fragaria* across the whole genome and for each chromosome using a combination of *D* statistic, *D*_FOIL_, and PhyloNet analyses.

The *D-*statistic analysis (ABBA-BABA test; Durand et al. 2011) requires a four-taxon topology in the form (((P1,P2),P3),O), where O is the outgroup, and P1 and P2 are ingroups that are tested for signals of gene flow with P3. In the outcome of the *D-*statistic analysis, a *Z*-score > 3 & *D*-score < 0 indicates gene flow between P1 and P3, while *Z*-score > 3 & *D*-score > 0 indicates gene flow between P2 and P3. In this study, we set *P. micrantha* as the outgroup and performed *D*-statistic analyses using the evobiR package in R (https://github.com/coleoguy/evobir) to detect the signals of introgression for 8,663 orthologs for all ten possible combinations of the four-taxon topology. The detail results are shown in Fig. 2d and Table S13.

The *D*_FOIL_ analysis (Pease et al. 2015) assumes a symmetrical five-taxon topology following (((P1,P2),(P3,P4)),O), where O is the outgroup, P1 to P4 are ingroups, and the divergence of P3 and P4 should be earlier than the divergence of P1 and P2. *D*_FOIL_ can also estimate the direction of gene flow. In this study, we applied the software *D*_FOIL_ (<https://github.com/jbpease/dfoil>) to examine the gene flow signals in two fixed-taxon topologies: (i), (((*F. nilgerrensis*, *F. nubicola*), (*F. viridis*, *F. vesca*)), *F. iinumae*) and (ii), (((*F. nilgerrensis*, *F. nubicola*), (*F. viridis*, *F. vesca*)), *P. micrantha*). The details are shown in Fig. 2e and Table S14.

The PhyloNet analysis (Wen et al. 2018) was developed to reconstruct reticulated phylogenies from gene trees or alignments without an underlying reference topology. Here, we used the MCMC_SEQ program (https://wiki.rice.edu/confluence/display/PHYLONET/MCMC_SEQ) withhin PhyloNet to identify signals for gene flow across the genus and displayed the results by using Dendroscope 3 (Huson and Scornavacca 2012) (Fig. 3f).

**Tracing diploid ancestors by using sppIDer**

SppIDer is a recently developed pipeline for elucidating the contributions of putative parental genomes to hybrids. This pipeline does not require phylogenetic reconstruction and, therefore, is free from the assumptions of phylogeny that are sometimes violated when used to infer the origins of hybrids. SppIDer maps short-read sequencing data simultaneously to a combination of multiple reference genomes, consisting of putative parental species, and assesses the genomic contribution of each to the hybrid. Still, sppIDER is useful for rapid inference of interspecies hybrids, even with missing reference genomes. Reads would map to the genome of the sister species, if it was available, or that they would fail to map or be distributed across other genomes, if there were no close relatives (Langdon et al. 2018).

In this study, we downloaded Illumina resequencing data from 73 accessions of cultivated octoploid strawberries (*F.* × *ananassa*) from the NCBI database (BioProject accession No.: PRJNA578384; Table S15). After quality control, we mapped short reads from each sample to the combined reference representing genomes of five diploid species of *Fragaria*: *F. nilgerrensis*, *F. nubicola*, *F. viridis*, *F. vesca*, and *F. iinumae*. Based on this mapping, we assessed contributions of each diploid to the commercial strawberry and displayed results using violin graphs (Fig. 3a). As an example, we visualized the depth of mappings at each locus of the combined reference using the octoploid strawberry sample, FL_13C026p134 (Accession No.: SRR10312160 & SRR10312161), which has highest coverage among the 73 samples (Fig. 3b; Fig. S7).

**Orthogroup clustering among commercial strawberry and the five dioploid *Fragaria* plants**

We used OrthoFinder (Emms and Kelly 2015) with the parameters *S* = diamond and *og* to classify proteins from the five diploid species of *Fragaria* and the cultivated octoploid strawberry (Edger et al. 2019). Further, we compared the genes that occur in the diploid species of *Fragaria* but are lacking in the cultivated strawberry. These genes comprised two categories: (i) the species-specific genes from each of the five diploid species of *Fragaria* and (ii) the genes existing in at least two diploid species of *Fragaria* but absent in the cultivated strawberry. The results are shown in Fig. 3c.

To better understand the functional roles of the genes that are absent in cultivated strawberry but exist in the diploid species, we annotated transcription factors and protein kinases, the resistance genes, and genes related to flowering time and fruit quality using iTAK (Zheng et al. 2016), RGAugury (Li et al. 2016), and KEGG Orthology (KO), respectively. The results are shown in Table S17.

**Reference**

Altschul SF, Gish W, Miller W, Myers EW, Lipman DJ. 2009. Basic local alignment search tool. J Mol Biol. 215: 403-410.

Birney E, Clamp M, Durbin R. 2004. GeneWise and Genomewise. Genome Res. 14: 988-995.

Bouckaert RR. 2010. DensiTree: making sense of sets of phylogenetic trees. Bioinformatics 26: 1372-1373.

Bru C, Courcelle E, Carrre S, Beausse Y, Dalmar S, Kahn D. 2005. The ProDom database of protein domain families: more emphasis on 3D. Nucleic Acids Res. 33: D212-215.

Burge C, Karlin S. 1997. Prediction of complete gene structures in human genomic DNA. J Mol Biol. 268: 78-94.

Burton JN, Adey A, Patwardhan RP, Qiu R, Kitaman JO, Shendure J. 2013. Chromosome-scale scaffolding of de novo genome assemblies based on chromatin interactions. Nat Biotechnol. 31, 1119–1125.

Darzentas N. 2010. Circoletto: visualizing sequence similarity with Circos. Bioinformatics 26: 2620-2621.

De Bie T, Cristianini N, Demuth JP, Hahn M. 2006. CAFE: a computational tool for the study of gene family evolution. Bioinformatics 22: 1269-1271.

Durand EY, Patterson N, Reich D, Slatkin M. 2011. Testing for ancient admixture between closely related populations. Mol Biol Evol. 28: 2239-2252.

Emms DM, Kelly S. 2015. OrthoFinder: solving fundamental biases in whole genome comparisons dramatically improves orthogroup inference accuracy. Genome Biol. 16: 157.

Feng C, Wang J, Wu L, Kong H, Yang L, Feng C, Wang K, Rausher M, Kang M. 2020. The genome of a cave plant, *Primulina huaijiensis*, provides insights into adaptation to limestone karst habitats. New Phytol. 227: 1249-1263.

Feng C, Xu MZ, Feng C, von Wettberg EJB, Kang M. 2017. The complete chloroplast genome of *Primulina* and two novel strategies for development of high polymorphic loci for population genetic and phylogenetic studies. BMC Evol Biol. 17: 224.

Grabherr MG, Haas BJ, Yassour M, Levin JZ, Thompson DA, Amit I, Adiconis X, Fan L, Raychowdhury R, Zeng QD, et al. 2011. Full-length transcriptome assembly from RNA-Seq data without a reference genome. Nat Biotechnol. 29: 644-652.

Guigó R, Knudsen S, Drake N, Smith TF. 1992. Prediction of gene structure. J Mol Biol. 226: 141-157.

Haas BJ, Delcher AL, Mount SM, Wortman JR, Smith RK, Hannick LI, Maiti R, Ronning CM, Rusch DB, Town CD, et al. 2003. Improving the *Arabidopsis* genome annotation using maximal transcript alignment assemblies. Nucleic Acids Res. 31: 5654-5666.

Haas BJ, Salzberg SL, Zhu W, Pertea M, Allen JE, Orvis J, White O, Buell CR, Wortman JR. Automated eukaryotic gene structure annotation using EvidenceModeler and the program to assemble spliced alignments. Genome Biol. 9: R7.

Hulo N, Bairoch A, Bulliard V, Cerutti L, De Castro E, Langendijk-Genevaux PS, Pagni M, Sigrist CJA. 2006. The PROSITE database. Nucleic Acids Res*.* 34: D227-230.

Huson DH, Scornavacca C. 2012. Dendroscope 3: An interactive tool for rooted phylogenetic trees and networks, Syst Biol. 61: 1061-1067.

Katoh K, Standley DM. 2013. MAFFT Multiple sequence alignment software version 7: improvements in performance and usability. Mol Biol Evol. 30: 772-780.

Kent WJ. 2002. BLAT-the BLAST-like alignment tool. Genome Res. 12: 656-664.

Kim D, Pertea G, Trapnell C, Pimentel H, Kelley R, Salzberg SL. 2013. TopHat2: accurate alignment of transcriptomes in the presence of insertions, deletions and gene fusions. Genome Biol*.* 14: R36.

Korf I. 2004. Gene finding in novel genomes. BMC Bioinformatics 5: 59.

Langdon QK, Peris D, Kyle B, Hittinger CT. 2018. sppIDer: a species identification tool to investigate hybrid genomes with high-throughput sequencing. Mol Biol Evol. 35: 2835-2849.

Li H. 2018. Minimap2: pairwise alignment for nucleotide sequences. Bioinformatics 34: 3094-100.

Li H, Durbin R. 2009. Fast and accurate short read alignment with Burrows-Wheeler Transform. Bioinformatics 25: 1754-1760.

Li PC, Quan XD, Jia GF, Xiao J, Cloutier S, You FM, 2016. RGAugury: a pipeline for genome-wide prediction of resistance gene analogs (RGAs) in plants. BMC Genomics 17: 852.

Majoros WH, Pertea M, Salzberg SL 2004. TigrScan and GlimmerHMM: two open source *ab initio* eukaryotic gene-finders. Bioinformatics 20: 2878-2879.

Mirarab S and Warnow T. 2015. ASTRAL-II: coalescent-based species tree estimation with many hundreds of taxa and thousands of genes. Bioinformatics 31: 44-52.

Mirarab S, Reaz R, Bayzid MS, Zimmermann T, Swenson MS, Warnow T. 2014. ASTRAL: genome-scale coalescent-based species tree estimation. Bioinformatics 30: I541-I548.

Mistry J, Finn R. 2007. Pfam: a domain-centric method for analyzing proteins and proteomes. Methods Mol Biol. 396: 43-58.

Myers EW. 2005. The fragment assembly string graph. Bioinformatics, 21: ii79-ii85.

Nguyen LT, Schmidt HA, von Haeseler A, Minh BQ. 2015. IQ-TREE: A fast and effective stochastic algorithm for estimating maximum-likelihood phylogenies. Mol Biol Evol*.* 32: 772-780.

Ou SJ, Jiang N. 2018. LTR_retriever: A highly accurate and sensitive program for identification of long terminal repeat retrotransposons. Plant Physiol. 176: 1410-1422.

Pease JB, Brown JW, Walker JF, Hinchliff CE, Smith SA. 2018. Quartet Sampling distinguishes lack of support from conflicting support in the green plant tree of life. Am J Bot. 105: 385-403.

Pease JB, Hahn MW. 2015. Detection and polarization of introgression in a five-taxon phylogeny. Syst Biol. 64: 651-662.

Roach MJ, Schmidt SA, Borneman AR. 2018. Purge Haplotigs: allelic contig reassignment for third-gen diploid genome assemblies. BMC Bioinformatics 19: 460.

Sanderson MJ. 2003. r8s: inferring absolute rates of molecular evolution and divergence times in the absence of a molecular clock. Bioinformatics 19: 662-684.

Schultz J, Milpetz F, Bork P, Ponting CP. 1998. SMART, a simple modular architecture research tool: identification of signaling domains. Proc Natl Acad Sci USA*.* 95:5857-5864.

Stamatakis A. 2014. RAxML version 8: a tool for phylogenetic analysis and post-analysis of large phylogenies. Bioinformatics 30: 1312-1313.

Stanke M, Schoffmann O, Morgenstern B, Waack S. 2006. Gene prediction in eukaryotes with a generalized hidden Markov model that uses hints from external sources. BMC Bioinformatics 7: 62.

Suyama M, Torrents D, Bork P. 2006. PAL2NAL: robust conversion of protein sequence alignments into the corresponding codon alignments. Nucleic Acids Res. 34: W609-W612.

Trapnell C, Williams BA, Pertea G, Mortazavi A, Kwan G, van Baren MJ, Salzberg SL, Wold BJ, Pachter L. 2010. Transcript assembly and quantification by RNA-Seq reveals unannotated transcripts and isoform switching during cell differentiation. Nat Biotechnol. 28: 511-515.

Waterhouse RM, Seppey M, Simao FA, Manni M, Ioannidis P, Klioutchnikov G, Kriventseva EV, Zdobnov EM. 2018. BUSCO applications from quality assessments to gene prediction and phylogenomics. Mol Biol Evol. 35: 543-548.

Wen D, Yu Y, Zhu J, Nakhleh L. 2018. Inferring phylogenetic networks using PhyloNet, Syst Biol*.* 67: 735-740.

Xiang Y, Huang C, Hu Y, Wen J, Li S, Yi T, Chen H, Xiang J, Ma H. 2016. Evolution of Rosaceae fruit types based on nuclear phylogeny in the context of geological times and genome duplication. Mol Biol Evol. 34: 262-281.

Xu Z, Wang H. 2007. LTR_FINDER: an efficient tool for the prediction of full-length LTR retrotransposons. Nucleic Acids Res. 35: W265-W268.

Yang Y, Smith SA. 2014. Orthology inference in nonmodel organisms using transcriptomes and low-coverage genomes: improving accuracy and matrix occupancy for phylogenomics. Mol Biol Evol. 31: 3081-3092.

Zdobnov EM, Apweiler R. 2001. InterProScan-an integration platform for the signature recognition methods in InterPro. Bioinformatics 17: 847-848.

Zheng Y, Jiao C, Sun HH, Rosli HG, Pombo MA, Zhang PF, Banf M, Dai XB, Martin GB, Giovannoni JJ, et al. 2016. iTAK: a program for genome-wide prediction and classification of plant transcription factors, transcriptional regulators, and protein kinases. Mol Plant. 9: 1667-1670.
