# Supplementary figures and images for: Tracing the Diploid Ancestry of the Cultivated Octoploid Strawberry
Source: Mol Biol Evol. 2020 Sep 17;38(2):478–85. doi: 10.1093/molbev/msaa238 (PMC7826170; doi:10.1093/molbev/msaa238)

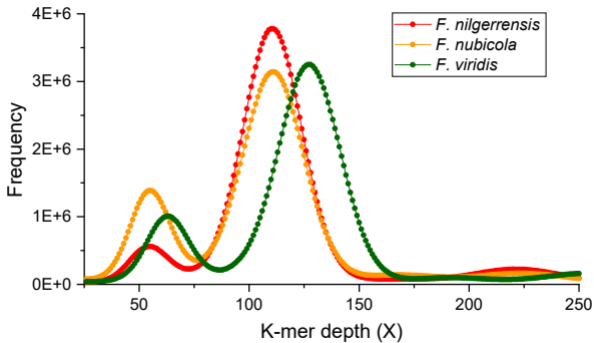

Supplement: msaa238_Supplementary_Data [file msaa238_supplementary_data.zip › msaa238-suppl_data/Figure S1.pdf]

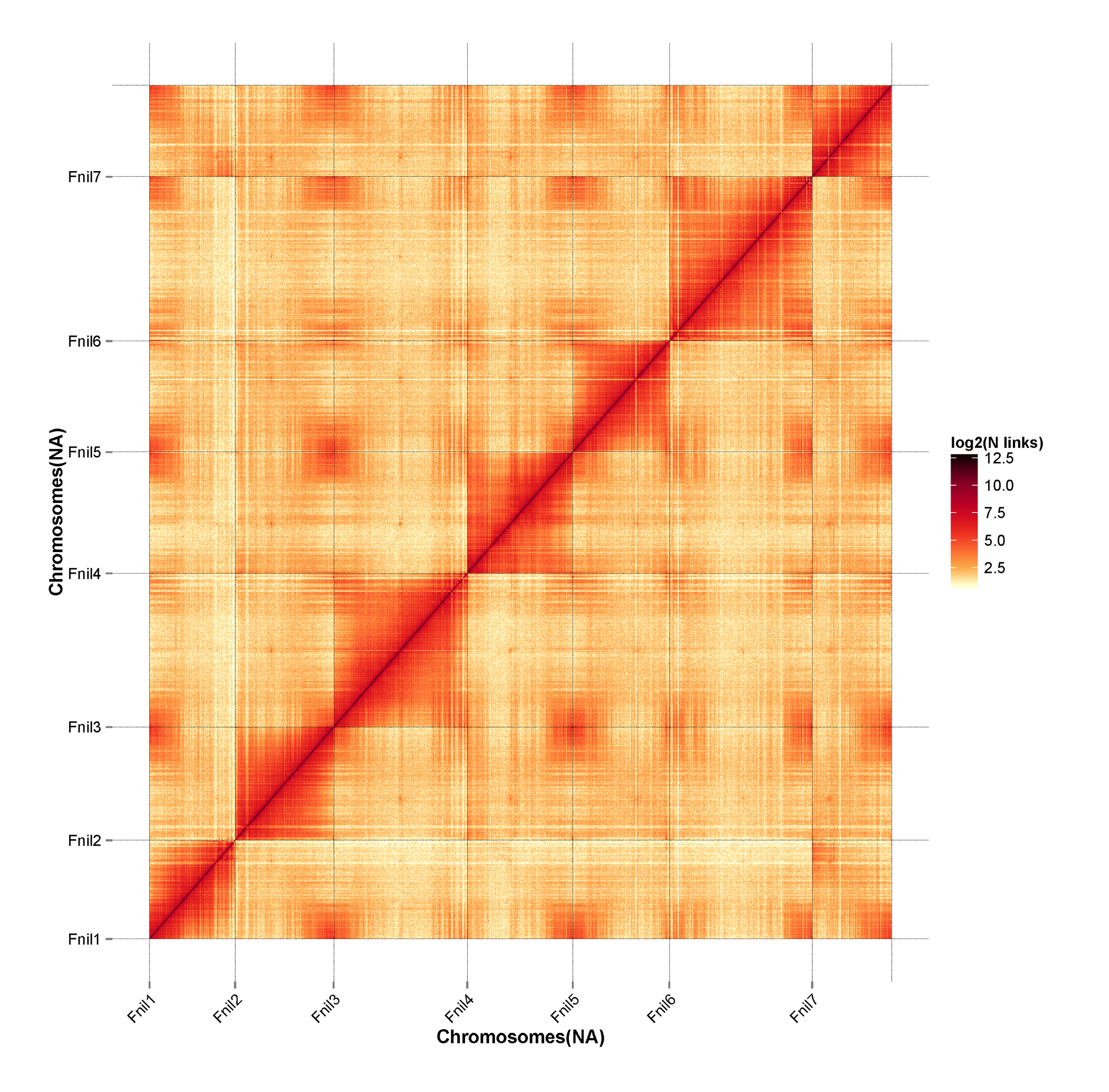

Supplement: msaa238_Supplementary_Data [file msaa238_supplementary_data.zip › msaa238-suppl_data/Figure S2.jpg]

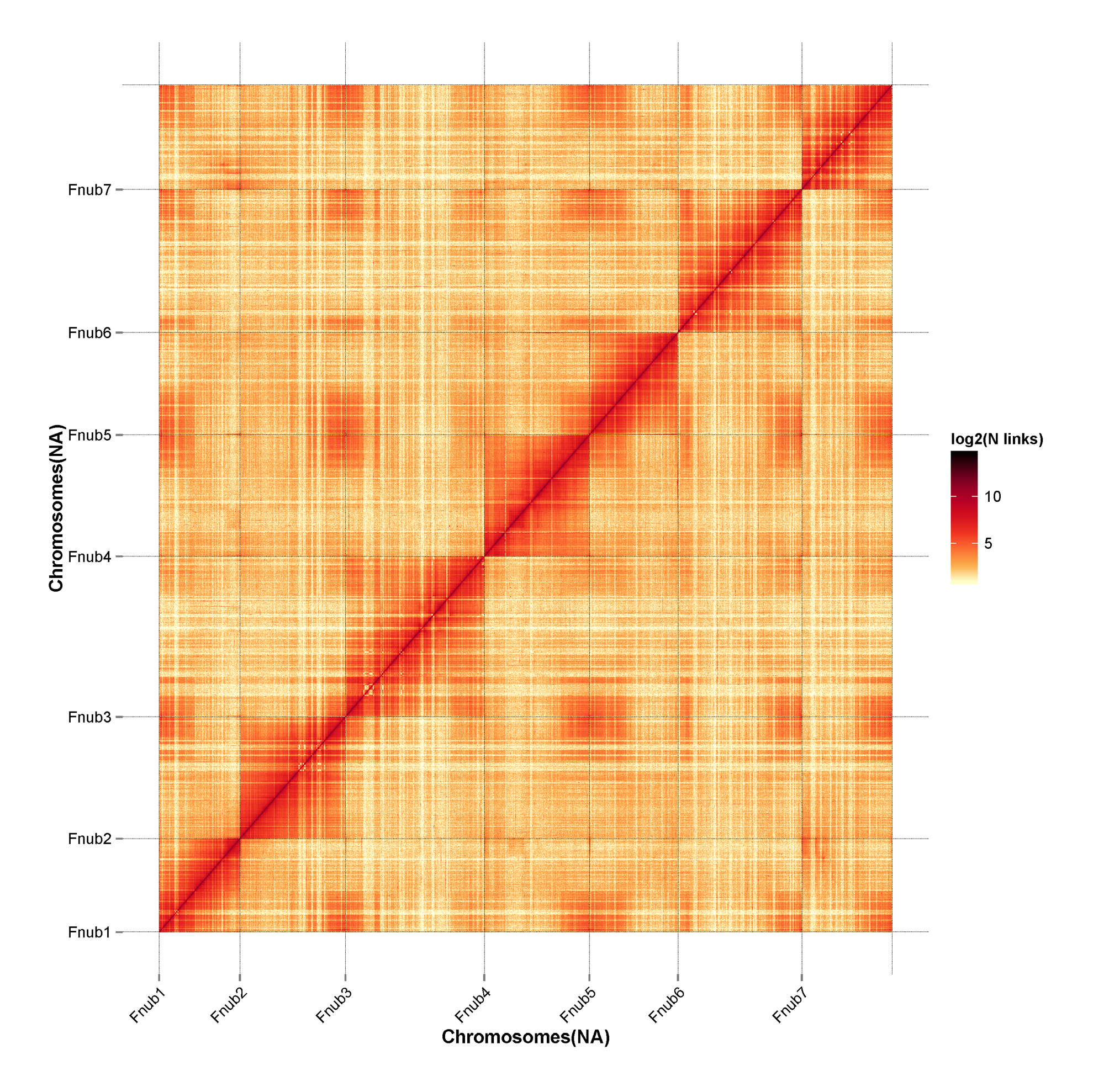

Supplement: msaa238_Supplementary_Data [file msaa238_supplementary_data.zip › msaa238-suppl_data/Figure S3.jpg]

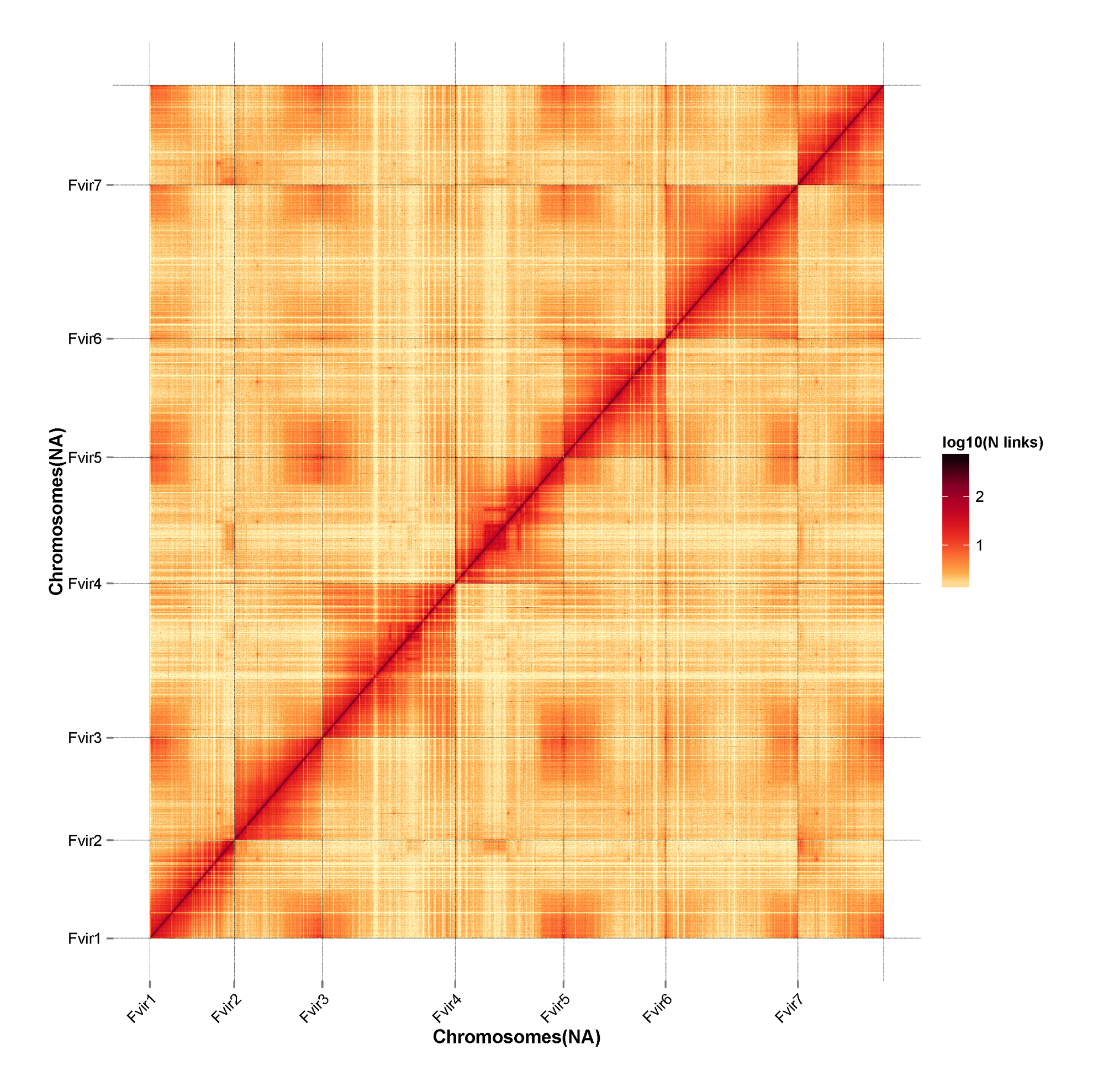

Supplement: msaa238_Supplementary_Data [file msaa238_supplementary_data.zip › msaa238-suppl_data/Figure S4.jpg]

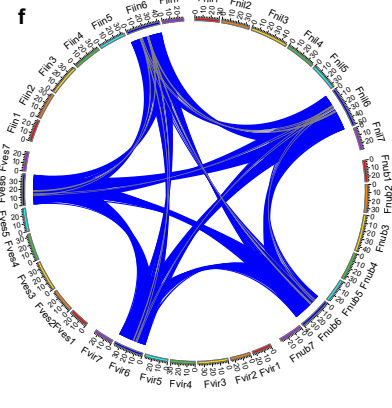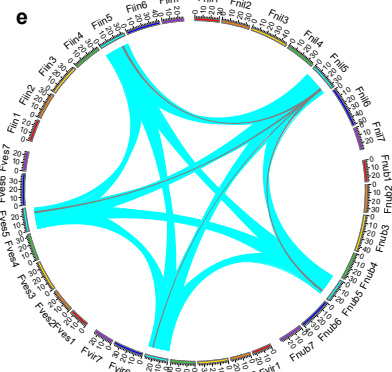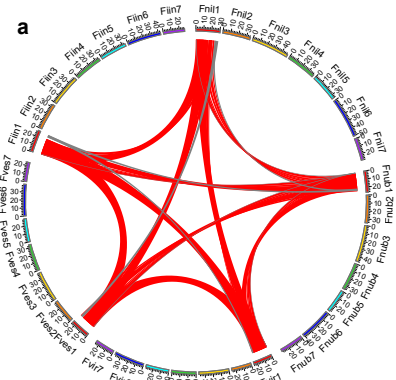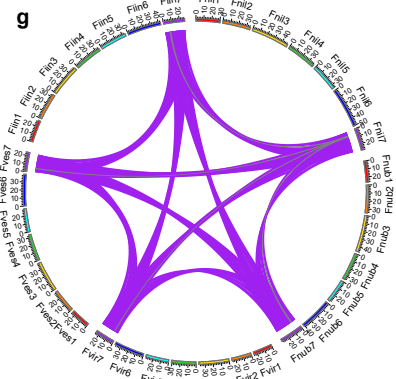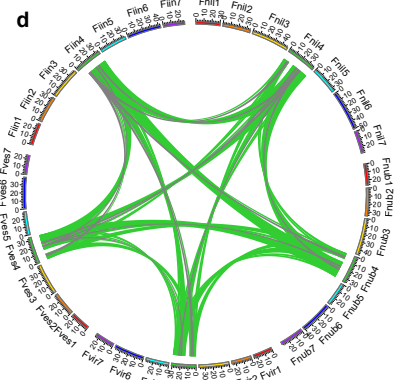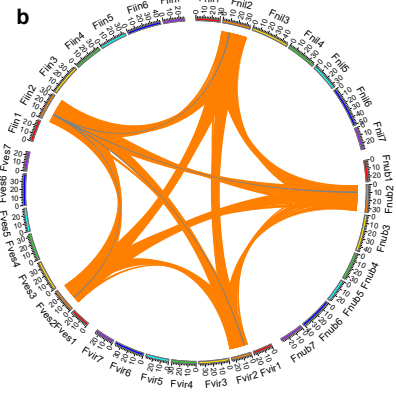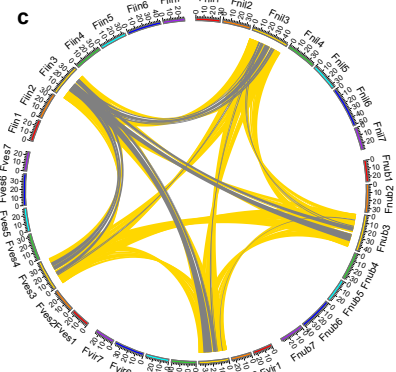

Supplement: msaa238_Supplementary_Data [file msaa238_supplementary_data.zip › msaa238-suppl_data/Figure S5.pdf]

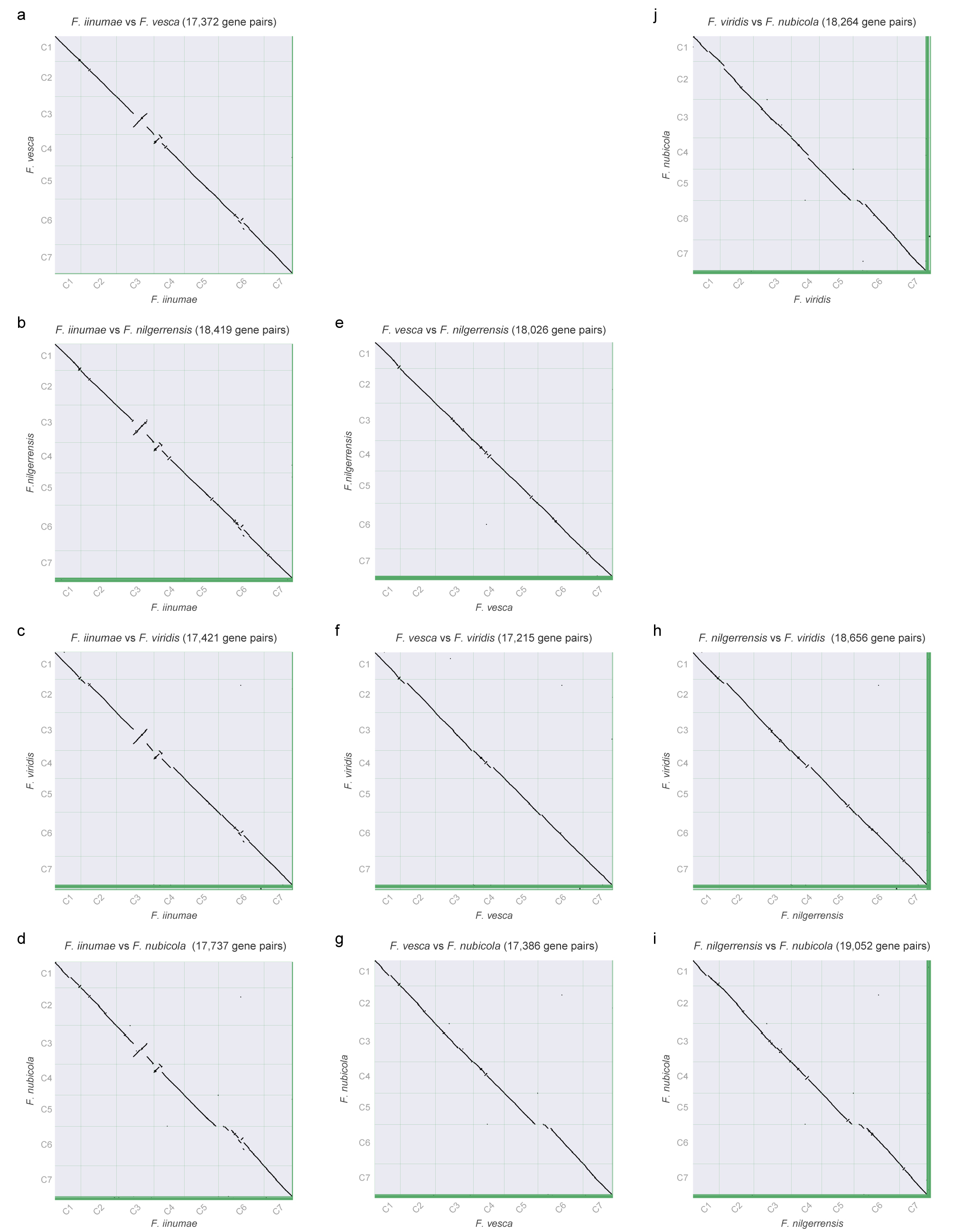

Supplement: msaa238_Supplementary_Data [file msaa238_supplementary_data.zip › msaa238-suppl_data/Figure S6.jpg]

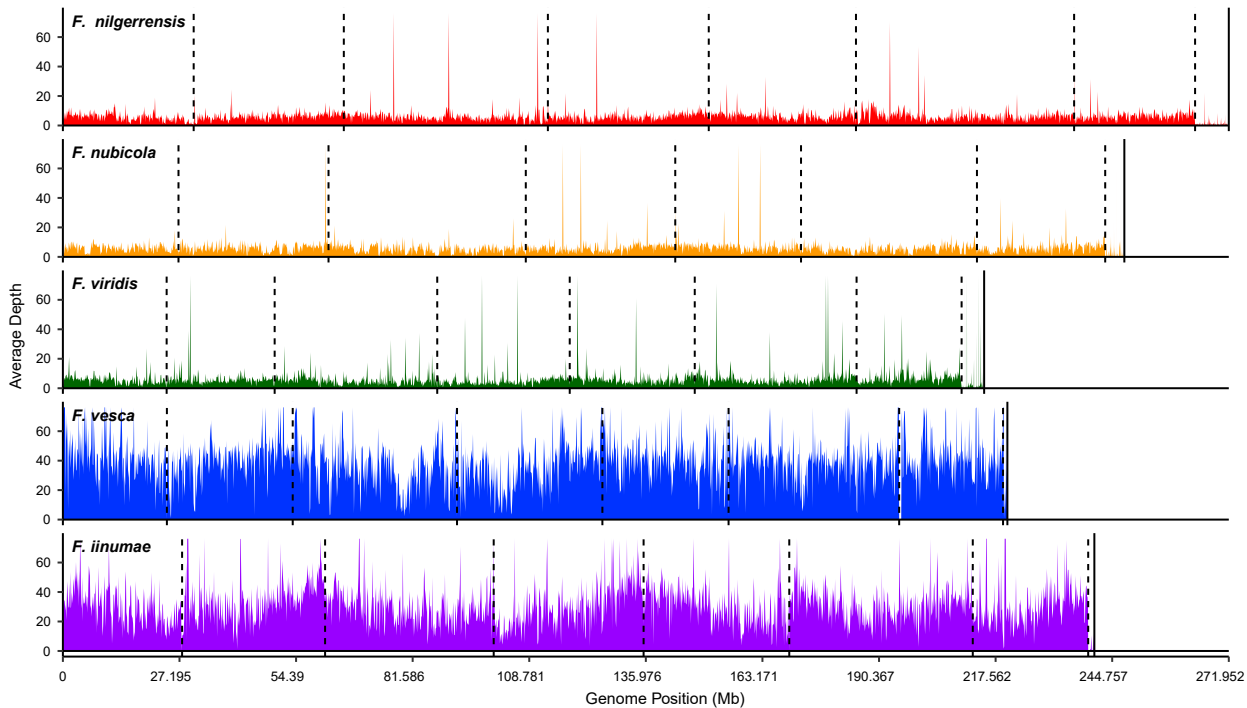

Supplement: msaa238_Supplementary_Data [file msaa238_supplementary_data.zip › msaa238-suppl_data/Figure S7.pdf]

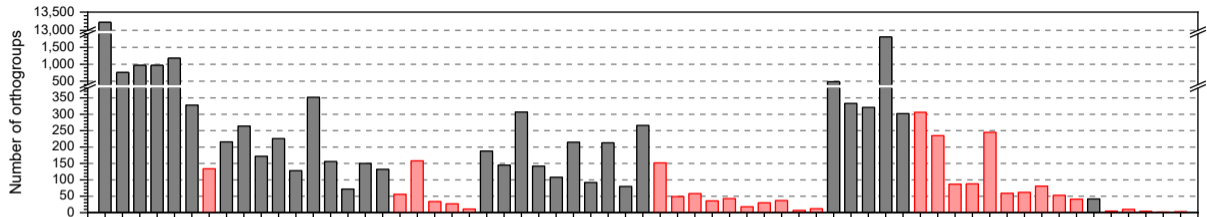

*F. x ananassa*

*F. nilgerrensis*

*F. nubicola*

*F. viridis*

*F. vesca*

*F. iinumae*

Core

Dispensable

Specific

Supplement: msaa238_Supplementary_Data [file msaa238_supplementary_data.zip › msaa238-suppl_data/Figure S8.pdf]
